# Supplementary material for: A Functional Network Driven by MicroRNA-125a Regulates Monocyte Trafficking in Acute Inflammation
Source: Int J Mol Sci. 2022 Sep 14;23(18):10684. doi: 10.3390/ijms231810684 (PMC9503790; doi:10.3390/ijms231810684)
Supplement: Supplementary file 1 [file ijms-23-10684-s001.zip › ijms-1900391-supplementary.pdf]

Supplementary Information

Table S1. The primer sequences.

Method	Name	Sequence
Cloning	JAM-A forward	5′ GTGTGTTGGAGGGCTTCTTAG 3′
	JAM-A reverse	5′ ATGCAGACCACCCATGATCT 3′
Cloning	JAM-L forward	5′ AGTCCCTTCATCTCAGCAGC 3′
	JAM-L reverse	5′ CAACCCATTCACCCCAAACC 3′
qRT-PCR	CCR2 forward	5′TGAGACAAGCCACAAGCTGA 3′
	CCR2 reverse	5′ TTCTGATAAACCGAGAACGAGAT 3′
qRT-PCR	JAM-A forward	5′AAGTTGTCCTGTGCCTACTCG 3′
	JAM-A reverse	5′ CGGTCCTCATAGGAAGCTGT 3′
qRT-PCR	JAM-L forward	5′ CATGTTTTGCCCACTGAAAC 3′
	JAM-L reverse	5′ CATTCAGGCCCAAGGAATAA 3′
qRT-PCR	B2M forward	5′TTCTGGCCTGGAGGCTATC 3′
	B2M reverse	5′ TCAGGAAATTTGACTTTCCATTC 3′
qRT-PCR	TBP forward	5′ GAACATCATGGATCAGAACAACA 3′
	TBP reverse	5′ ATAGGGATTCCGGGAGTCAT 3′

Supplementary Figure S1

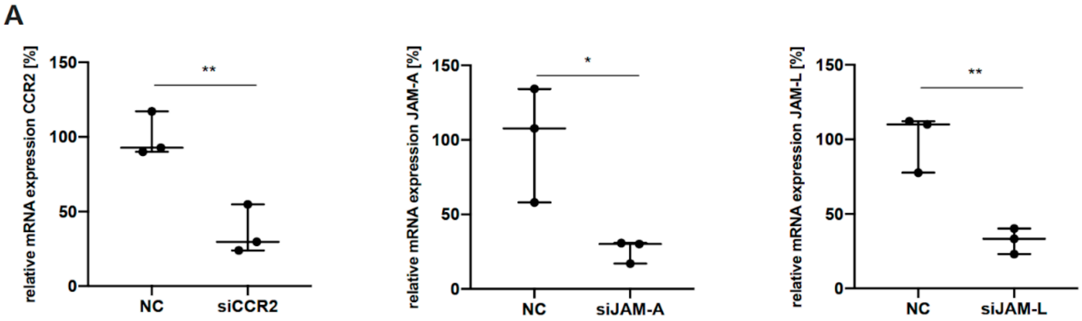

Supplementary Figure S1 Knockdown efficiency of primary human monocytes transfected with siRNAs targeting CCR2, JAM-A and JAM-L.

(**A**) Knockdown efficiency of siRNAs indicated as relative mRNA expression of respective target mRNA after transfection of individual siRNAs compared to negative control (n=3, *$p < 0.05$, **$p < 0.01$).

Supplementary Figure S2. Gene names of regulated genes on the heatmap ordered by clusters.

Cluster 1

11726524_s_at – ZFHX3
11727707_a_at – BBC3
11742871_a_at – PIK3IP1
11748808_a_at – ARID3B
11720739_s_at – ITPKB
11751393_a_at – PIK3IP1
11741051_x_at – IMPDH1
11729130_at – ANAPC16
11725297_a_at – MAPRE2
11737440_a_at – BMF
11720768_at – METTL9
11730935_a_at – AKNA
11740932_a_at – CSF3R
11723521_x_at – ZNF385A
11749329_a_at – ARHGAP9
11721421_s_at – RAP1GAP2
11734778_a_at – PCTP
11750840_s_at – TGFBR2
11717720_a_at – ST6GAL1
11735157_at – CYSLTR1
11737504_x_at – SIRPB2
11756441_x_at – CSF3R
11727552_at – FZD2
11749377_a_at – CECR1
11741447_s_at – RNASE4
11755975_x_at – PKIB
11743171_a_at – RCSD1
11763426_a_at – TRAF3IP3
11727643_s_at – TRERF1
11742816_a_at – FAM13A
11738980_a_at – CD4
11743170_a_at – RCSD1
11721583_x_at – LRMP
11760710_a_at – MS4A6A
11731066_x_at – GIMAP1
11737820_a_at – MAP3K3
11724061_s_at – OSBPL1A
11763695_a_at – ABTB1
11725830_a_at – PLCL2
11739079_a_at – ANP32A
11734777_a_at – PCTP
11729013_at – ACPP
11718818_s_at – SLC26A6
11721107_a_at – TRABD
11734920_s_at – PCYOX1L
11758673_s_at – SORD
11726393_at – FGD4
11750157_at – CD4
11752633_a_at – OLFM1
11723885_at – NATD1
11754362_a_at – TTC7A
11727385_a_at – PCCA
11730331_at – BRI3BP
11745554_a_at – ASGR1
11724292_s_at – TRIT1
11718283_a_at – EIF4EBP1
11732135_a_at – ADCK3
11756746_s_at – NA
11739744_x_at – MXI1
11757868_a_at – PIK3IP1
11757767_s_at – NAV1
11754492_x_at – PLCB2
11720595_a_at – CD46
11725092_s_at – RFX5
11742219_a_at – CD46
11751532_x_at – CD46
11731200_a_at – TLR5
11758893_at – MLXIP
11727007_at – SORT1
11753491_s_at – ERP29
11718284_x_at – EIF4EBP1

[illegible]
11720057_a_at – POLR1D
11733389_a_at – DEF6
11724081_a_at – TOR2A
11724082_x_at – TOR2A
11756694_a_at – RNF44
11743343_at – RMND5A
11724499_at – MFNG
11755502_x_at – TECR
11718676_x_at – TMEM245
11763731_a_at – VPS36
11722039_x_at – FES
11756983_a_at – F11R
11758645_s_at – EIF4EBP2
11755355_s_at – RCSD1
11730478_at – RNF125
11753321_a_at – NA
11752590_s_at – CCR2
11723849_a_at – MS4A6A
11718062_a_at – NME3
11718675_s_at – TMEM245
11744475_x_at – TRABD
11729748_a_at – IL10RA
11745326_x_at – DUS1L
11716402_at – EIF4EBP2
11738091_a_at – ABTB1
11722888_s_at – NA
11756846_a_at – PRAM1
11722038_a_at – FES
11740874_a_at – FES
11755715_x_at – CD46
11750001_x_at – CLEC7A
11751549_a_at – CD46
11722952_a_at – SLC27A1
11720461_a_at – F11R
11720763_a_at – SELPLG
11729758_at – RGS18
11728559_a_at – ASGR1
11717341_at – PTGFRN
11719667_s_at – STMN1
11718988_s_at – RNASE4
11738982_at – CD4
11738981_x_at – CD4
11724979_a_at – DPEP2
11757026_x_at – SERPINF1
11757025_s_at – SERPINF1
11724008_a_at – ENTPD1
11722970_a_at – CREB5
11722971_a_at – CREB5
11733801_s_at – CPPED1
11753705_x_at – STMN1
11732351_at – HGF
11722681_at – RBP7
11752825_a_at – ARHGEF6
11735309_a_at – MYCL
11728522_a_at – MYCL
11751425_x_at – TRAF3IP3
11736681_at – SLC46A1
11755091_a_at – ABCA7
11724291_a_at – TRIT1
11721582_a_at – LRMP
11721569_a_at – ANG
11746729_a_at – LRMP
11745165_a_at – TRAF3IP3
11759029_at – BRI3BP
11751424_a_at – TRAF3IP3
11749685_a_at – FAM13A
11750052_a_at – ASGR2
11722448_at – KCNMB4
11759152_at – CYSLTR1
11749188_a_at – ENTPD1
11724992_x_at – GTPBP3

11743378_a_at – NIN
11723433_at – TBC1D22A
11751525_a_at – CCDC69
11730323_at – RPS29
11740088_s_at – PIK3IP1
11717339_s_at – SORD
11752942_s_at – DBP
11716232_at – CTNNBIP1
11736962_a_at – MBNL2
11758238_s_at – ABCC4
11749411_a_at – SPIDR
11720856_a_at – INSR
11746542_x_at – SPIDR
11747171_a_at – MXD4
11718304_s_at – NA
11748699_a_at – RGS14
11735728_x_at – ZNF385A
11725091_at – RFX5
11720464_x_at – F11R
11751394_s_at – PIK3IP1
11742872_s_at – PIK3IP1
11759028_at – BRI3BP
11744281_a_at – FAM53B
11749056_a_at – RCSD1
11730477_at – RNF125
11718081_a_at – ATP2B4
11743879_at – MAML3
11719332_at – ARHGAP15
11750682_a_at – TPCN1
11725093_a_at – RFX5
11741179_a_at – ABTB1
11749057_x_at – RCSD1
11749214_a_at – SELPLG
11741250_a_at – SH2D3C
11747498_s_at – SORD
11729268_a_at – STAC3
11740018_a_at – INSR
11754685_a_at – RGS14
11754990_a_at – EPB41
11755873_a_at – ACSS1
11727553_a_at – PKIB
11737038_at – C19orf38
11743677_at – P2RY8
11717508_at – IRF4
11731147_at – TLR7
11756371_a_at – VSIG4
11731148_at – TLR7
11762274_x_at – HLA–DMB
11725827_s_at – CD1D
11729871_a_at – ASGR2
11760018_at – CLEC10A
11727554_s_at – PKIB
11726485_at – CPED1
11741874_x_at – SEPP1

Cluster 2

11753719_a_at – GMFB
11753358_a_at – ACVR1
11758185_s_at – TWF1
11746929_a_at – MOB3C
11735676_a_at – OSGIN1
11717524_s_at – RDX
11748968_x_at – RDX
11724523_a_at – CDK5RAP2
11728498_a_at – SVIL
11745431_a_at – SVIL
11728499_x_at – SVIL
11722593_s_at – BCAP29
11757628_s_at – TSPYL2
11717843_at – ETNK1
11752386_a_at – NAB1
11730052_a_at – SNX16
11716945_s_at – TWF1
11746201_a_at – NAB1
11743972_a_at – DDIT4
11734657_s_at – NA
11745021_a_at – MYC
11721308_at – PHLDA1
11758781_at – EMP1
11744648_s_at – AIFM2
11733240_at – IRAK2
11757558_s_at – LONRF1
11729751_a_at – LPAR1
11724524_x_at – CDK5RAP2
11716654_a_at – EML4
11716653_x_at – EML4
11716500_a_at – PIR
11763806_x_at – KCNN4
11721307_at – PHLDA1
11729750_a_at – LPAR1
11715711_a_at – AKR1C3
11721775_s_at – ASPH
11745104_a_at – PJA1
11724715_a_at – PJA1
11748596_a_at – KLHL2
11757836_s_at – SEC24A
11763581_a_at – SLC30A6
11724676_a_at – KLHL2
11731452_s_at – SEC62
11720444_s_at – ERLIN1
11748951_a_at – CTNNB1
11748952_x_at – CTNNB1
11747388_x_at – CTNNB1
11747361_x_at – HMGA1
11750680_x_at – CTNNB1
11723135_s_at – SRC
11720755_at – STAT3
11746889_a_at – CKAP4
11733110_s_at – TLK1
11736217_at – MAP3K8
11747602_a_at – MAP3K8
11749022_x_at – CTNNB1
11720769_a_at – PPIF
11717716_at – FNDC3B
11724424_at – GPR68
11717567_a_at – NQO1
11718875_a_at – PNMA1
11733109_a_at – TLK1
11737748_a_at – GMFB
11718218_at – RAB22A
11736513_s_at – NMT2
11736006_at – MAPK1IP1L
11732718_at – EREG
11717715_at – FNDC3B
11722796_a_at – MAPK6
11763785_x_at – KCNN4
11719227_x_at – KCNN4
11729847_a_at – CCL7

Cluster 3

11744921_a_at – SLC43A1
11756191_a_at – MAN1C1
11750879_a_at – CREB5
11738889_at – IQCD
11720857_a_at – INSR
11728286_at – RBMS2
11746198_a_at – METTL9
11751817_x_at – MBNL2
11725410_at – NAV1
11737027_a_at – FLT3
11748399_s_at – VCL
11721626_a_at – SEPT6
11746773_a_at – SLC27A1
11719036_at – UVRAG
11725053_x_at – TOP1MT
11755265_s_at – NT5DC1
11741251_a_at – SIRPB2
11731879_a_at – NLRP1
11747618_a_at – ADCK3
11748695_a_at – SYNGR1
11735306_at – C9orf139
11733650_s_at – FAM228B
11755804_a_at – SEMA4D
11730068_a_at – HGF
11740366_a_at – SHMT1
11753045_a_at – MBNL2
11732417_at – FAM105A
11720204_at – NT5DC1
11728285_at – RBMS2
11732699_a_at – PKIB
11732700_x_at – PKIB
11758583_s_at – TMEM154
11724763_s_at – DEPTOR
11737134_x_at – ZNF385A
11731602_at – TMEM154
11752189_a_at – ARHGEF6
11728961_a_at – NA
11754521_a_at – MBNL2
11734791_x_at – SEPT6
11742063_a_at – BTN3A1
11753046_x_at – MBNL2
11724293_a_at – TRIT1
11739770_s_at – TMOD2
11759015_at – SPECC1
11747732_a_at – ADCK3
11753704_a_at – STMN1
11751748_a_at – CHN2
11727642_a_at – TRERF1
11719666_a_at – STMN1
11729227_a_at – GRK5
11716403_at – EIF4EBP2
11730843_a_at – MXI1
11731578_s_at – ACSS1
11749445_a_at – ARHGAP15
11726459_at – FAM117B
11762132_a_at – AMICA1

Cluster 4

11722391_at - MTF1
11725045_a_at - UAP1
11749608_a_at - RNF11
11753001_a_at - PPARG
11758864_at - SRP19
11736274_at - TLR2
11718139_at - PDXK
11759869_x_at - PJA1
11746814_x_at - NAB1
11739508_a_at - NAA30
11721330_s_at - RNGTT
11739746_s_at - SVIL
11717383_at - FOPNL
11761935_at - HNRNPLL
11730914_a_at - ERLIN1
11763822_at - EREG
11747839_a_at - LPAR1
11717527_a_at - RDX
11729235_s_at - E2F6
11725276_a_at - CDK5RAP2
11731077_a_at - LONRF1
11731076_a_at - LONRF1
11763594_a_at - HS3ST3B1
11739367_at - ASPH
11757312_x_at - SERPINE1
11726809_x_at - SLC12A8
11724275_s_at - TMEM158
11735268_s_at - SLC41A2
11727186_a_at - WNT5A
11734811_a_at - EGR2
11735116_a_at - MET
11721774_a_at - ASPH
11731577_a_at - SLC22A1
11715751_a_at - DPYSL3
11737251_at - CD226
11745300_a_at - DNAJC6
11758577_s_at - ELOVL7
11727189_x_at - WNT5A
11751610_a_at - DPYSL3
11717376_a_at - SLC25A25
11724915_at - TMTC3
11746856_a_at - SERPINE1
11735724_a_at - TP53BP1
11718808_a_at - TP53BP1
11752291_x_at - SLC30A6

Cluster 5

11759611_at - SMIM11A
11724007_s_at - ENTPD1
11725568_a_at - ATP8A1
11751656_a_at - TMEM154
11721955_at - RFX7
11756758_x_at - SPECC1
11758230_s_at - NFIA
11749401_a_at - TRIT1
11732747_at - HGF
11716390_a_at - BACE1
11724625_a_at - SPATA7
11756155_a_at - NHS
11724762_a_at - DEPTOR

Cluster 6

[illegible]
11728984_a_at - MTHFD1L
11756610_a_at - CD226
11727188_at - WNT5A
11719215_a_at - NA
11727840_at - HPD
11752813_a_at - MSC-AS1
11745301_a_at - DNAJC6

Cluster 7

11715635_x_at - TMEM14C
11744429_a_at - STX10
11719780_at - TNFAIP8L2
11739119_s_at - CNPY3
11739146_a_at - MKNK2
11739720_a_at - GIMAP8
11757612_a_at - ASRGL1
11732589_a_at - ZNF467
11742220_x_at - CD46
11716633_a_at - TMEM50A
11719025_a_at - ZNF652
11716404_s_at - EIF4EBP2
11751550_x_at - CD46
11718610_at - TP53INP1
11722767_s_at - MBNL2
11723692_a_at - CD302
11718090_a_at - TPCN1
11732418_at - FAM105A
11729759_at - RGS18
11719845_a_at - SMAP2
11747948_a_at - SMAP2
11720219_a_at - MAP3K3
11739118_a_at - CNPY3
11719157_x_at - ASRGL1
11717903_s_at - IER3IP1
11723712_x_at - MLXIP
11742744_a_at - STX10
11733402_a_at - CLEC7A
11754976_x_at - CNPY3
11732415_s_at - TIAM1
11745553_s_at - SMAP2
11720218_a_at - MAP3K3
11730936_s_at - AKNA
11730498_s_at - MXI1
11737870_s_at - FAM78A
11743771_at - GIMAP7
11757602_x_at - ERP29
11759405_x_at - STX10
11743903_a_at - CD46
11735060_a_at - CD46
11744364_s_at - INPP5D
11717618_s_at - PXN
11723368_a_at - ARHGEF6
11726947_a_at - TLR8
11717340_at - PTGFRN
11718002_at - C10orf54
11715816_at - ZFP36L2
11740238_a_at - SULF2
11720460_x_at - F11R
11716527_a_at - GSTK1
11721159_x_at - DRAM2
11715627_x_at - ALDH2
11717762_a_at - CPVL
11749293_x_at - MS4A6A
11730996_a_at - AMICA1
11728523_a_at - MYCL
11752737_a_at - CLEC7A
11733403_x_at - CLEC7A
11727788_a_at - PIGS
11741510_a_at - HERPUD1
11730383_a_at - SNX18
11716359_a_at - FAM96A
11735681_x_at - CD46
11726820_a_at - ARHGAP9
11718971_x_at - CD46
11720380_a_at - IMPDH1
11742681_s_at - ERP29
11743931_x_at - ERP29
11752331_s_at - NA
11736017_s_at - PXN
11721702_a_at - CMTM7
11719844_at - SMAP2

[illegible]
11750531_a_at - PYCARD
11721169_a_at - PYCARD
11730090_a_at - NA
11716554_a_at - HLA-DMA
11745989_a_at - HLA-DMA
11757815_s_at - METTL7A
11723691_at - CD302
11752948_a_at - PRCP
11721158_a_at - DRAM2
11729747_a_at - IL10RA
11726948_at - TLR8
11715848_a_at - ATP5G2
11718160_at - KLF13
11754471_a_at - HLA-DMB
11750769_s_at - CCR2
11716846_a_at - MS4A6A
11746962_x_at - HLA-DMB
11735352_x_at - SLC37A2
11731676_s_at - CCR2
11740531_a_at - CLEC10A
11745500_a_at - CLEC10A
11746961_a_at - HLA-DMB
11750856_s_at - CCR2
11749803_s_at - ARL8B
11754863_s_at - VPS37C

Cluster 8

11749803_s_at – ARL8B
11754863_s_at – VPS37C
11745421_a_at – BID
11755603_a_at – ARL8B
11749021_s_at – CTNNB1
11731110_at – ADAM17
11716718_at – GLA
11720458_at – CAPRIN1
11756634_a_at – GSR
11721502_at – SWAP70
11719354_s_at – SH3BP5
11736273_a_at – TLR2
11747640_a_at – TDP2
11743066_s_at – PTDSS1
11722031_s_at – RNF11
11747482_a_at – HNRNPR
11726583_s_at – CKAP4
11721801_at – ARL8B
11742819_at – PLAUR
11718648_s_at – ACVR1
11722705_at – B3GNT5
11720771_x_at – PPIF
11722728_a_at – EGR2
11744680_a_at – SLC7A11
11739475_s_at – NAB1
11752899_s_at – SEC62
11764019_s_at – NAB1
11753204_a_at – PTDSS1
11719368_s_at – MAPKAPK2
11717326_at – KLF9
11721448_a_at – ETF1
11747387_a_at – CTNNB1
11717526_a_at – RDX
11720370_x_at – RTCA
11729163_a_at – BTG3
11717525_s_at – RDX
11721306_at – PHLDA1
11739366_at – ASPH
11717582_a_at – RGCC
11717566_at – EMP1
11719355_s_at – SH3BP5
11721305_a_at – PHLDA1
11720770_a_at – PPIF

Cluster 9

[illegible]
11741350_a_at – ANXA11
11757303_s_at – BNIP3L
11741103_a_at – ANXA11
11754666_a_at – CORO1A
11742944_a_at – CORO1A
11718001_a_at – C10orf54
11715817_at – ZFP36L2
11715818_at – ZFP36L2
11754681_s_at – HLA–DMB
11717012_s_at – VAMP8
11716377_s_at – GRN
11757420_s_at – ZFP36L2
11748543_a_at – TXNIP
11759666_x_at – HLA–DPB1
11724682_s_at – TGFBR2
11756073_x_at – HLA–DPB1
11756393_x_at – CORO1A

Cluster 10

[illegible]
11750538_x_at – TXN
11753775_x_at – TXN
11731454_a_at – SEC62
11721876_s_at – SLC7A11
11720772_s_at – PPIF
11753804_x_at – TXN
11715470_x_at – TXN

Cluster 11

[illegible]
11756431_s_at – TXNIP
11759642_x_at – HLA–DRB1

Cluster 12

[illegible]
11731450_s_at – SEC62
11719819_s_at – EMC10
11759080_at – GSR
11755523_a_at – RBM47
11722167_s_at – CTNNB1
11754833_a_at – TLR2
11740390_a_at – RBM47
11721802_s_at – ARL8B
11715649_s_at – TXNRD1
11721875_at – SLC7A11
11717565_s_at – EMP1
11754026_a_at – CXCL8
11763226_x_at – CXCL8
